# Effect of nonpharmacologic therapies on depressive symptoms in patients with chronic fatigue syndrome: a network meta-analysis

**DOI:** 10.3389/fpsyt.2025.1657615

**Published:** 2025-08-19

**Authors:** Baiyi Jiang, Mengru Cao, Xue Xia, Long Wang

**Affiliations:** ^1^ Department of Graduate School, Heilongjiang University of Chinese Medicine, Harbin, China; ^2^ Department of Medicine IV, The Tumour Hospital of Harbin Medical University, Harbin, China; ^3^ Department of Acupuncture and Moxibustion, The First Affiliated Hospital of Heilongjiang University of Chinese Medicine, Harbin, China

**Keywords:** depression, non-pharmacological therapy, network meta-analysis, systematic review, chronic fatigue syndrome

## Abstract

**Background:**

Depression or depressive symptoms exacerbate the burden in patients with chronic fatigue syndrome (CFS). The therapeutic effects of various non-pharmacological interventions remain unclear.

**Objective:**

This paper aims to evaluate the effectiveness of different non-pharmacological measures in alleviating depression or depressive symptoms in patients with CFS through network meta-analysis.

**Methods:**

PubMed, Cochrane Library, Web of Science, Embase, CNKI, Wanfang, CBM, VIP, and Sinomed databases were searched for randomized controlled trials (RCTs) until March 26, 2025. The Cochrane Risk of Bias Assessment Tool 2.0 was utilized to appraise the risk of bias. A network meta-analysis was conducted using the GeMTC package in R (4.4.2). This protocol has been registered in PROSPERO (CRD420251020737).

**Results:**

47 RCTs involving 4,028 participants were included. Compared with control measures, diet therapy was most effective in improving depression or depressive symptoms in patients with CFS (SMD = -5.64, 95% CI: -8.98 to -2.29), followed by moxibustion (Mox) (SMD = -2.91, 95% CI: -4.61 to -1.22), acupuncture (Ap) + Mox + acupoint embedding (SMD = -3.16, 95% CI: -0.39 to -5.98), and Ap + Mox (SMD = -2.53, 95% CI: -1.17 to -3.91).

**Conclusion:**

Diet therapy is the most effective in improving depression or depressive symptoms in patients with CFS, followed by Mox. Further carefully designed RCTs are warranted to substantiate these findings.

**Systematic review registration:**

https://www.crd.york.ac.uk/PROSPERO/, identifier CRD420251020737.

## 1 Introduction

Chronic fatigue syndrome (CFS) is a chronic disease that affects multiple systems and has complex and diverse symptoms, usually manifested as persistent fatigue and pain that cannot be relieved by rest and worsens after exercise (1). The prevalence of CFS is approximately 0.3%-3.3%. Due to the diversity of diagnoses, the actual number of affected people in the United States may be much higher. Emotional problems such as anxiety and depression are widespread among CFS patients (2). Depression is an affective disorder characterized primarily by pronounced and persistent low mood, with symptoms coexisting across emotional, physical, and cognitive dimensions (3). Depression and CFS have many similarities in symptoms and have only been clearly divided into two diseases in recent years, with a distinct bidirectional relationship (4). Michael et al. (5) found that CFS patients had greatly elevated plasma levels of pro-inflammatory cytokines, which may induce or exacerbate depressive symptoms. Compared with CFS without depression, CFS with depression may cause more severe endocrine disorders (6). In terms of psychological cognition, maladaptive perfectionism is associated with depression levels in CFS patients (7). Doctors’ disregard for fatigue in CFS patients and illegal diagnosis and treatment are also factors contributing to comorbid depression (8). CFS patients suffer from severe physical and social functional impairment, face unemployment and economic crisis, and have an enhanced risk of depression (9). Depression is one of the independent risk factors for CFS, exacerbating the symptoms and functional impairment of chronic diseases. For every one-point increase in the depression scale score, the overall risk of CFS increases by 49% (10). CFS with concomitant depression impairs patients’ social functioning, significantly undermining their quality of life and increasing disability and suicide rates, imposing a substantial burden on individuals, the economy, and the healthcare system. It represents an urgent issue that requires resolution (11).

There is no specific drug for CFS, and symptomatic treatment is the main approach. When depressive symptoms appear, analgesics and antidepressants are often used. However, the effectiveness of these drugs is controversial, and their adverse reactions and addiction are obvious (12). Even clinical and animal studies have indicated that certain types of antidepressants and analgesics enhance CFS risk (13, 14). A large body of evidence suggests that non-pharmacological therapies can effectively alleviate depressive symptoms of CFS. For example, cognitive behavioral therapy (CBT) can alleviate fatigue symptoms and improve depression in CFS patients (15, 16). Acupuncture (Ap) and moxibustion (Mox) can improve physical and mental fatigue symptoms and psychological status in CFS patients (17). B. A. Gordon et al. (18) found that graded exercise considerably improved physical function, quality of life, and depressive symptoms in CFS patients. In this study, we only considered non-pharmacological therapies.

In addition to efficacy, non-pharmacological therapies have several advantages over pharmacological therapies, including fewer side effects, high tolerability, low cost, increased patient compliance, favorable doctor-patient interaction, personalized regimens, and no restrictions on medical resources (19). The European consensus on diagnosis and treatment released in 2024 recommends that when CFS is secondary to depression, pacing therapy and psychotherapy should be the priority for treatment (20). The guidelines issued by the American College of Physicians also recommend that CBT be given priority in the treatment of depression (21), which suggests that non-pharmacological therapies may have obvious advantages in the treatment of CFS patients with depressive symptoms. However, in this field, the evaluation of many non-pharmacological therapies such as Ap and exercise lacks high-quality evidence, and the effectiveness of some therapies is controversial. For example, Internet-based CBT relieved symptoms in CFS patients with comorbid depressive symptoms by about 20% compared with non-depressed patients (22). Exercise therapy may exceed the patient’s energy limit, causing characteristic post-exertional malaise (PEM) and affecting efficacy (23). Nevertheless, there is currently a lack of comparative studies on the efficacy of non-pharmacological therapies in improving depressive symptoms in CSF, This paper employs a network meta-analysis (NMA) method to systematically search Chinese and English databases for studies related to non-pharmacological therapies for depressive symptoms in CFS patients, to comprehensively analyze current non-pharmacological therapies in this field. It has the advantage of integrating direct and indirect comparisons using existing research while comparing multiple interventions, to investigate the efficacy of non-pharmacological treatments for depressive symptoms in CFS patients. This, to a certain extent, fills the gap in clinical research, provides a basis for non-pharmacological treatment options for clinical treatment of depressive symptoms in CSF patients, and offers references and suggestions for public health decisions and clinical guidelines.

## 2 Methods

This study followed the PRISMA guidelines and their requirements for NMA (24). The study protocol was registered in the PROSPERO (CRD420251020737).

### 2.1 Search strategy

PubMed, Cochrane Library, Embase, Web of Science, CNKI, VIP, Wanfang, and Sinomed databases were searched from establishment date to March 26, 2025, and the language was restricted to Chinese and English. The search was conducted using a combination of subject terms and free terms. The medical subject terms included “Fatigue Syndrome”, “Chronic Fatigue Syndrome”; “Depression”, “Depressive Disorder”, “Central Depression”; and “randomized controlled trial”. References in other relevant articles and gray literature were manually searched to pinpoint studies that met the criteria. The specific search strategy used is listed in **Supplementary Table 1**.

### 2.2 Inclusion and exclusion criteria

Literature meeting the criteria were included: (1) Study subjects: CSF patients; (2) Interventions: any non-pharmacological treatment; with conventional care and sham AP as control interventions; (3) Study type: randomized controlled trial (RCT); (4) Outcome measures: depression or depressive symptoms, assessed using scales, including PROMIS Depression Short Form (PROMIS Depression-SF), Hospital anxiety and depression scale (HADS), Beck Depression Inventory (BDI), Patient Health Questionnaire-9 (PHQ-9), SPHERE, Self-rating depression scale (SDS), Symptom Checklist-90 (SCL-90), Brief Symptom Inventory (BSI), and Hamilton Depression Scale (HAMD), and Depression Status Inventory (DSI).

The following types of literature were ruled out: (1) animal or cell experiments, case reports, reviews, scientific experimental plans, letters, editorials, and conference papers; (2) literature with missing data or serious errors; (3) duplicated publications; (4) literature without full text; (5) literature with overlapping study participants; (6) literature with no data or data that cannot be extracted; (7) literature with irrelevant intervention measures; (8) Non-English or non-Chinese language.

### 2.3 Data extraction

The retrieved literature was imported into EndNote, and two researchers (Baiyi Jiang and Xue Xia) independently screened the titles and abstracts and then read the full text for a secondary screening. For literature with discrepancies, re-evaluation was conducted after discussion or consultation with a third researcher (Long Wang). Data extraction was performed independently by two researchers using a pre-designed electronic form, including first author, publication year, country, randomization and blinding design, intervention and control measures, treatment duration, basic information of study subjects, and outcome measures.

### 2.4 Quality assessment

Two authors (Baiyi Jiang and Xue Xia) independently used the Cochrane risk of bias tool (ROB 2.0) to appraise the quality of eligible studies (25) in five aspects: random sequence generation, allocation concealment, blinding implementation, missing data, and selective reporting. Each aspect was rated as “high risk”, “some concerns”, or “low risk”. If all five items were low risk or only one item was rated as some risk and the rest were rated as low risk, the article was considered low risk. If four or more of the five items were rated as some risk or any one item was high risk, the article was considered high-risk. In all other cases, the article was considered moderate risk. The two authors independently completed the literature quality assessment, and any discrepancies were reviewed by a third author (Long Wang).

### 2.5 Statistical analysis

All outcome measures included were continuous variables. Since the scales used for outcome measurements varied across studies, the standardized mean difference (SMD) was adopted as the effect size measure. This study employed a Markov chain Monte Carlo method to construct a Bayesian NMA model and iterated the model to estimate the relative efficacy of different treatment regimens (26). When using non-informative priors, it is assumed that all values within the confidence range of the result are equally likely to occur, and only data from the included studies are used in the analysis (27, 28). Using noninformative priors avoids introducing subjectivity and/or nonrandomized data into the analysis models. During the verification process, four model chains, annealing of 10,000, iteration of 50,000, detection step size of 10, and initial value of 2.5 were set to obtain posterior distribution. The NMA was implemented based on r transitivity, homogeneity, and consistency assumptions. Heterogeneity was examined using the mtc:anohe function in the GeMTC package. When the overall I^2^ < 50%, the heterogeneity among the included studies in the same comparison was viewed as acceptable, satisfying the homogeneity assumption. The node splitting method was used to test the inconsistency between direct and indirect comparisons using the mtc.nodesplit function in the GeMTC package. When P > 0.05, it indicated no inconsistency between the direct and indirect comparisons, satisfying the consistency assumption. The convergence of the results was judged by calculating the potential scale reduction factor (PSRF), with 1 as the standard, and 1 ≤ PSRF < 1.05 indicated successful convergence. A network structure was constructed with SMDs between intervention measures as the line and each intervention measure as the node. The lines represent head-to-head comparisons among interventions. The surface area under the cumulative ranking curve (SUCRA) was estimated. Funnel plots were drawn to assess possible publication bias. All statistical analyses were made using R (version 4.4.2) and STATA (version 17) software.

## 3 Results

### 3.1 Literature search and screening process

**Figure 1:**
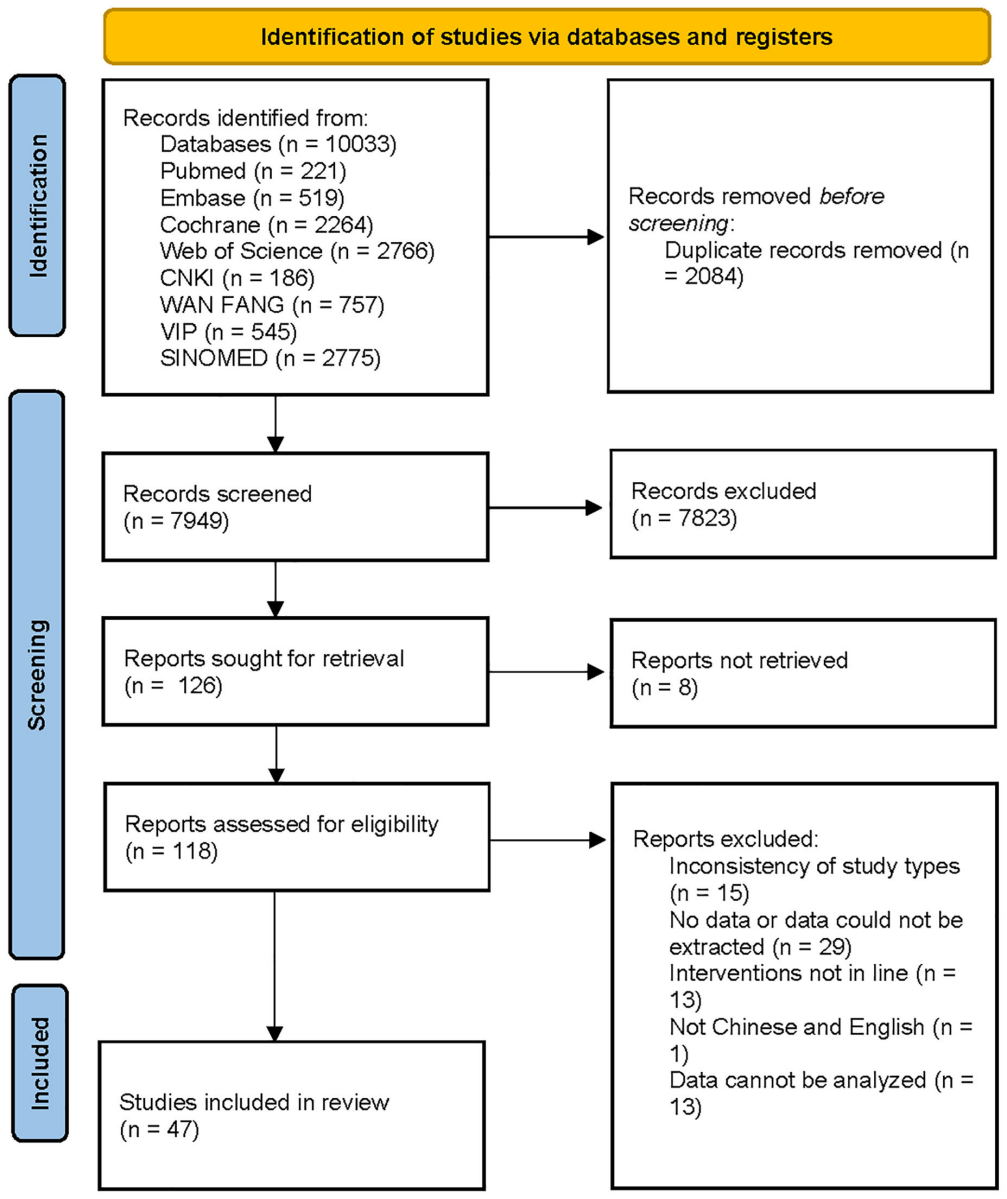
Literature search and screening flowchart.

10,033 documents were retrieved, of which 2,084 duplicates were eliminated. After preliminary reading of the titles and abstracts, 7,823 documents were excluded. Based on the secondary screening of the full text, documents were included or excluded strictly. Finally, 47 RCTs were included. The screening process is displayed in **Figure 1**.

### 3.2 Basic characteristics and quality assessment

The 47 included RCTs were from 10 countries (America, Australia, China, Netherlands, Britain, Germany, Portugal, Finland, Mexico, and Spain), involving 4,028 patients (1,246 males and 2,707 females) (sex data were sourced from 45 studies, and 2 studies did not report sex), aged 16–74 years. Interventions included AP, Mox, Cupping (Cup), Tuina, music therapy (MT), electroacupuncture, relaxation therapy, psychotherapy (Psy), aerobics (AER), diet therapy, resistance training (RT), Qigong, electrical stimulation, physiotherapy, Ap + Mox, Ap + Mox + Acupoints embedding (Ae), Psy + AER, Mox + RT, Mox + MT, and Ap + Mox + Cup. The control interventions included standard care, sham AP, sham transcranial direct current stimulation (tDCS), waiting list, no treatment, sham diathermy, health education, and sham chocolate. Basic characteristics of the included studies are summarized in **Table 1**.

**Table 1:** Basic information for included studies.

Authors	Year	Study Design	Area	Interventions	Sample Size	Sex (male/female)	Ages	Treatment Duration	Follow-up Time	Outcomes
J. F. K. El Mokadem	2023	RCT	America	Psychotherapy	11	3/19	43.45 ± 12.60	8 W	10 M	PROMIS Depression-SF
				Control	11		42.36 ± 8.57	8 W	/	PROMIS Depression-SF
KE. Wallman	2004	RCT	Australia	Aerobics	32	5/27	16-74	12 W	4 W	HADS
				Relaxation therapy	29	9/20	16-74	12 W	4 W	HADS
F. F. Xie	2022	RCT	China	Qigong	45	17/28	37.94 ± 11.34	12 W	/	HADS
				Psychotherapy	44	19/25	37.34 ± 9.86	12 W	/	HADS
J. S. Chan	2014	RCT	China	Qigong	22	3/19	43.60 ± 7.59	5 W	3 M	HADS
				Control	24	3/21	44.06 ± 4.88	5 W	3 M	HADS
M. Worm Smeitink	2019	RCT	Netherlands	Psychotherapy	121	52/69	37.20 ± 12.30	/	/	BDI
				Control	121	47/74	38.70 ± 12.50	/	/	BDI
T. Sathyapalan	2010	RCT	Britain	Diet therapy	10	4/6	52.00 ± 8.00	8W	/	HADS
				Control	10	4/6	52.00 ± 8.00	8W	/	HADS
P. Windthorst (60)	2017	RCT	Germany	Psychotherapy	13	0/13	51.40 ± 8.10	8 W	5 M	PHQ-9
				Aerobics	11	0/11	50.00 ± 10.9	8 W	5 M	PHQ-9
B. A. Gordon (18)	2010	RCT	Australia	Aerobics	11	/	16.20 ± 0.80	4 W	/	BDI
				Resistance-training	11	/	15.6 ± 1.6	4 W	/	BDI
T. Ma	2022	RCT	China	Ap+Mox	138	29/109	27.99 ± 3.24	8 W	12 W	SDS
				Acupuncture	138	25/113	27.67 ± 2.62	8 W	12 W	SDS
J. Li	2015	RCT	China	Qigong	22	3/19	43.60 ± 7.59	17 W	3 M	HADS
				Control	24	3/21	44.06 ± 4.88	17 W	3 M	HADS
A. Janse	2018	RCT	Netherlands	Psychotherapy	80	26/54	36.60 ± 12.80	27 W	6 M	SCL-90
				Control	80	35/45	39.90 ± 12.90	26 W	6 M	SCL-90
A. Janse	2016	RCT	Netherlands	Psychotherapy	50	18/32	37.61 ± 10.03	6 M	/	SCL-90
				Control	50	14/36	32.58 ± 9.81	6 M	/	SCL-90
F. Friedberg	2016	RCT	America	Psychotherapy	44	3/41	46.99 ± 10.79	3 M	12 M	BDI
				Control	48	6/42	50.03 ± 11.28	3 M	12 M	BDI
J. S. Chan	2013	RCT	HongKong	Qigong	72	20/52	42.40 ± 6.70	17 W	/	HADS
				Control	65	12/53	42.50 ± 6.40	17 W	/	HADS
M. Marques	2015	RCT	Portugal	Aerobics	45	1/44	46.96 ± 10.39	12 W	/	BSI
				Control	46	1/45	49.20 ± 11.49	12 W	/	BSI
L. Ridsdale	2012	RCT	Britain	Aerobics	71	15/56	42.60 ± 1.75	16 W	12 M	HADS
				Psychotherapy	76	18/58	39.70 ± 1.75	16 W	12 M	HADS
				Control	75	15/60	37.30 ± 1.50	16 W	12 M	HADS
L. Huanan	2017	RCT	China	Tuina	39	22/17	41.80 ± 7.10	4 W	3 M	HAMD
				Acupuncture	38	24/14	42.63 ± 6.20	4 W	3 M	HAMD
J. S. Chan	2017	RCT	China	Qigong	46	0/46	39.48 ± 2.67	9 W	3 M	HADS
				Control	62	0/62	41.66 ± 3.12	9 W	3 M	HADS
S.S. Chen	2018	RCT	China	Acupuncture	30	13/17	40.77 ± 11.61	4 W	/	SCL-90
				Control	30	7/23	41.47 ± 12.21	4 W	/	SCL-90
G. Deng	2013	RCT	America	Acupuncture	47	8/39	53.64 ± 2.70	6 W	6 M	HADS
				Control	50	9/41	52.82 ± 3.12	6 W	6 M	HADS
K.Y. Xue (17)	2023	RCT	China	Moxibustion	30	10/20	31.00 ± 7.25	13 D	14 D	SPHERE
				Acupuncture	30	8/22	28.00 ± 8.00	13 D	14 D	SPHERE
Z.X. Li	2022	RCT	China	Ap+Mox+Ae	30	12/18	33.87 ± 9.36	4 W	1 M	HAMD
				Acupuncture	30	14/16	34.25 ± 9.18	4 W	1 M	HAMD
S. H. Zheng	2013	RCT	China	Ap+Mox	30	9/21	42.00 ± 6.00	4 W	/	DSI
				Acupuncture	29	10/19	43.00 ± 6.00	4 W	/	DSI
G.X. An	2014	RCT	China	Acupuncture	42	18/24	36.49 ± 4.12	4 W	/	DSI
				Control	38	17/21	37.08 ± 4.69	4 W	/	DSI
S. H. Zheng	2012	RCT	China	Acupuncture	39	15/24	38.73 ± 4.11	4 W	/	DSI
				Control	38	16/22	37.08 ± 5.32	4 W	/	DSI
J. X. Li	2017	RCT	China	Electrical stimulation	46	18/28	39.00 ± 10.00	4 W	/	SPHERE
				Control	43	17/26	38.00 ± 9.00	4 W	/	SPHERE
L.L. Wu	2009	RCT	China	Music therapy	30	12/18	38.63 ± 11.49	4 M	/	SCL-90
				Control	30	10/20	38.66 ± 10.94	4 M	/	SCL-90
Q. Hu	2016	RCT	China	Tuina	30	17/13	35.14 ± 3.51	38 D	/	DSI
				Acupuncture	30	15/15	36.14 ± 4.23	38 D	/	DSI
J. Zheng	2024	RCT	China	Ap+Mox	30	16/14	37.28 ± 4.12	34 D	/	SDS
				Acupuncture	30	13/17	36.12 ± 2.27	34 D	/	SDS
T. Jing	2015	RCT	China	Tuina	27	/	19.30 ± 3.06	12 W	/	SCL-90
				Mox+TN	26	/	20.46 ± 3.39	12 W	/	SCL-90
X. J. Wang	2022	RCT	China	Acupuncture	38	10/28	31.10 ± 8.70	3 W	/	DSI
				Ap+Mox	36	8/28	28.60 ± 8.70	3 W	/	DSI
X. Y. Qing	2024	RCT	China	Control	35	18/17	38.03 ± 6.15	4 W	/	SDS
				Cupping	35	16/19	38.56 ± 6.24	4 W	/	SDS
J. Ma	2022	RCT	China	Resistance-training	54	35/19	39.48 ± 8.35	4 W	/	SDS
				Mox+RT	54	37/17	39.52 ± 8.62	4 W	/	SDS
M.M. Wang	2021	RCT	China	Control	34	9/25	43.76 ± 7.56	4 W	/	SPHERE
				Mox+MT	33	6/27	42.51 ± 6.91	4 W	/	SPHERE
Y. F. Lin	2020	RCT	China	Control	28	6/22	34.00 ± 9.00	4 W	/	SDS
				Moxibustion	29	5/24	35.00 ± 9.00	4 W	/	SDS
Z.Q.Li	2021	RCT	China	Cupping	47	25/22	41.61 ± 4.29	2 M	/	SDS
				Ap+Mox+Cup	58	31/27	41.56 ± 4.23	2 M	/	SDS
L. Yang	2022	RCT	China	Tuina	150	73/77	38.10 ± 5.43	12 W	/	SDS
				Moxibustion	150	64/86	34.20 ± 6.12	12 W	/	SDS
X. Li	2021	RCT	China	Psychotherapy	30	6/24	37.33 ± 7.41	12 W	/	SDS
				Ap+Mox	30	8/22	35.53 ± 8.09	12 W	/	SDS
J. Zhao	2020	RCT	China	Control	29	17/12	45.60 ± 9.70	4 W	4 W	BDI
				Acupuncture	29	14/15	42.30 ± 10.20	4 W	4 W	BDI
P. Lappalainen	2024	RCT	Finland	Psychotherapy	50	8/42	45.90 ± 8.12	14 W	3 M	PHQ-9
				Control	53	6/47	46.30 ± 7.57	14 W	3 M	PHQ-9
S. Oliver Mas	2023	RCT	Mexico	Electrical stimulation	23	8/15	47.26 ± 9.05	2 W	1 M	BDI
				Control	24	2/22	44.12 ± 9.83	2 W	1 M	BDI
C. V. M. Sarmento	2020	RCT	America	Qigong	10	0/10	42.60 ± 10.70	10 W	/	HADS
				Control	10	0/10	56.10 ± 12.30	10 W	/	HADS
E. Ubeda D Ocasar	2024	RCT	Spanish	diathermy	15	0/15	53.67 ± 8.13	4 W	15 D	HADS
				Control	12	0/12	48.75 ± 10.90	4 W	15 D	HADS
T. T. Ma	2019	RCT	China	Ap+Mox	51	11/40	38.10 ± 13.50	8 W	/	SDS
				Acupuncture	61	9/52	37.43 ± 13.10	8 W	/	SDS
H. L. Luo	2011	RCT	China	Acupuncture	25	14/11	34.43 ± 8.70	1 Y	/	SDS
				Control	25	12/13	34.44 ± 8.97	1 Y	/	SDS
Y. H. Chu	2008	RCT	China	Electroacupuncture	30	6/24	38.5 ± 7.89	2 W	3 M	SPHERE
				Control	30	10/20	37.67 ± 9.85	2 W	3 M	SPHERE
F. J. Qi	2020	RCT	China	Tuina	30	17/13	35.14 ± 3.51	20 D	/	DSI
				Acupuncture	30	15/15	36.14 ± 4.23	20 D	/	DSI

Ae, Acupoints embedding; AER, Aerobics; AP, Acupuncture; BDI, Beck Depression Inventory; Cup, Cupping; DSI, Depression Status Inventory; HADS, Hospital anxiety and depression scale; HAMD, Hamilton Depression Scale; Mox, Moxibustion; MT, Music therapy; PHQ-9, Patient Health Questionnaire-9; PROMIS Depression-SF, PROMIS Depression Short Form; Psy, Psychotherapy; RT, Resistance training; SCL-90, Symptom Checklist-90; SDS, Self-rating depression scale.

**Figure 2:**
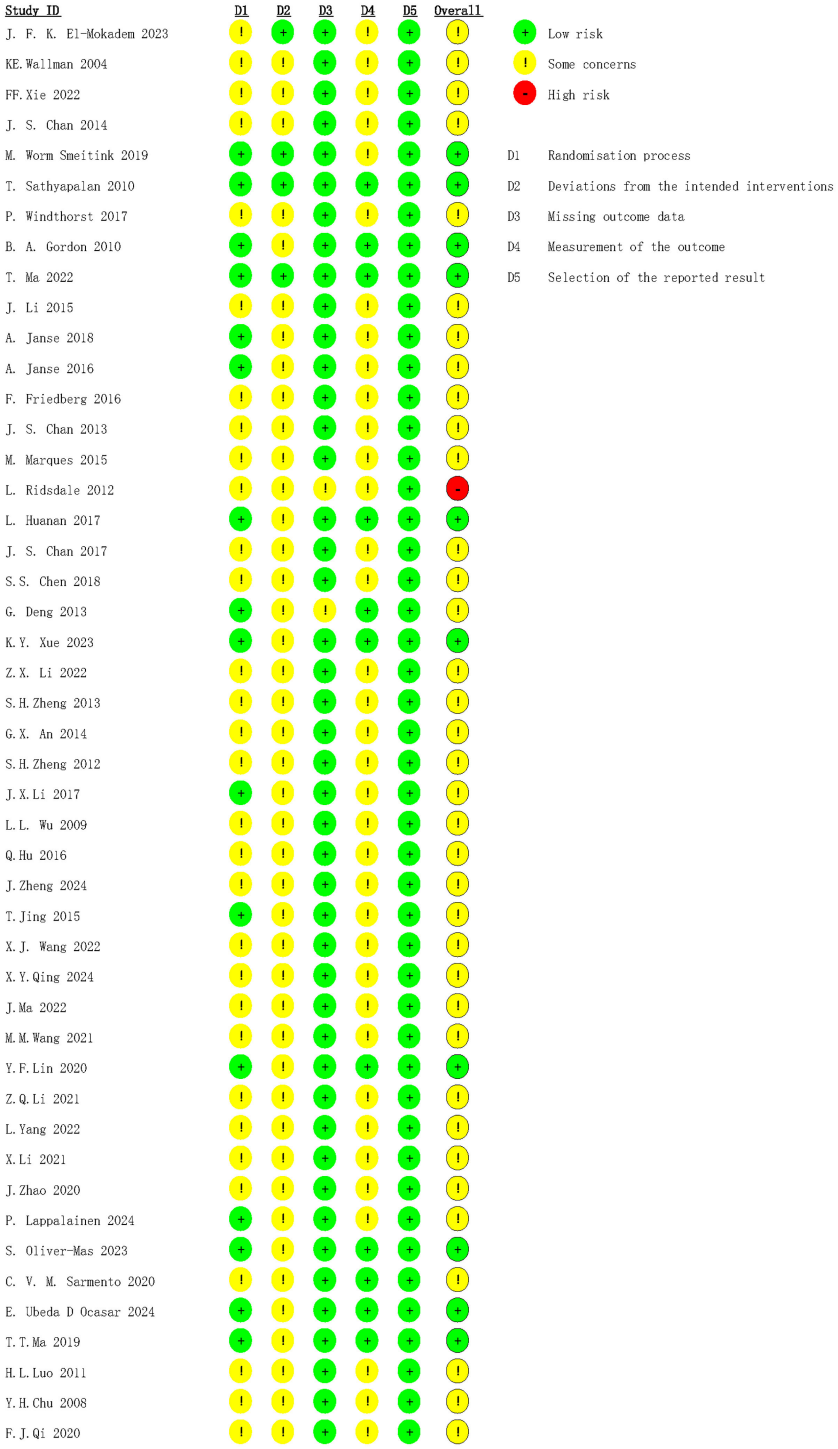
Risk of bias assessment.

The bias risk assessment showed that 10 RCTs were rated as low risk, and most studies had some concerns. The reasons for moderate risk were the absence of blinding and intention-to-treat analysis. One study was rated as high risk because baseline data of patients included differed greatly, and there were deviations from conventional medical care during the study (**Figure 2**).

### 3.3 NMA results

#### 3.3.1 Network diagram

**Figure 3:**
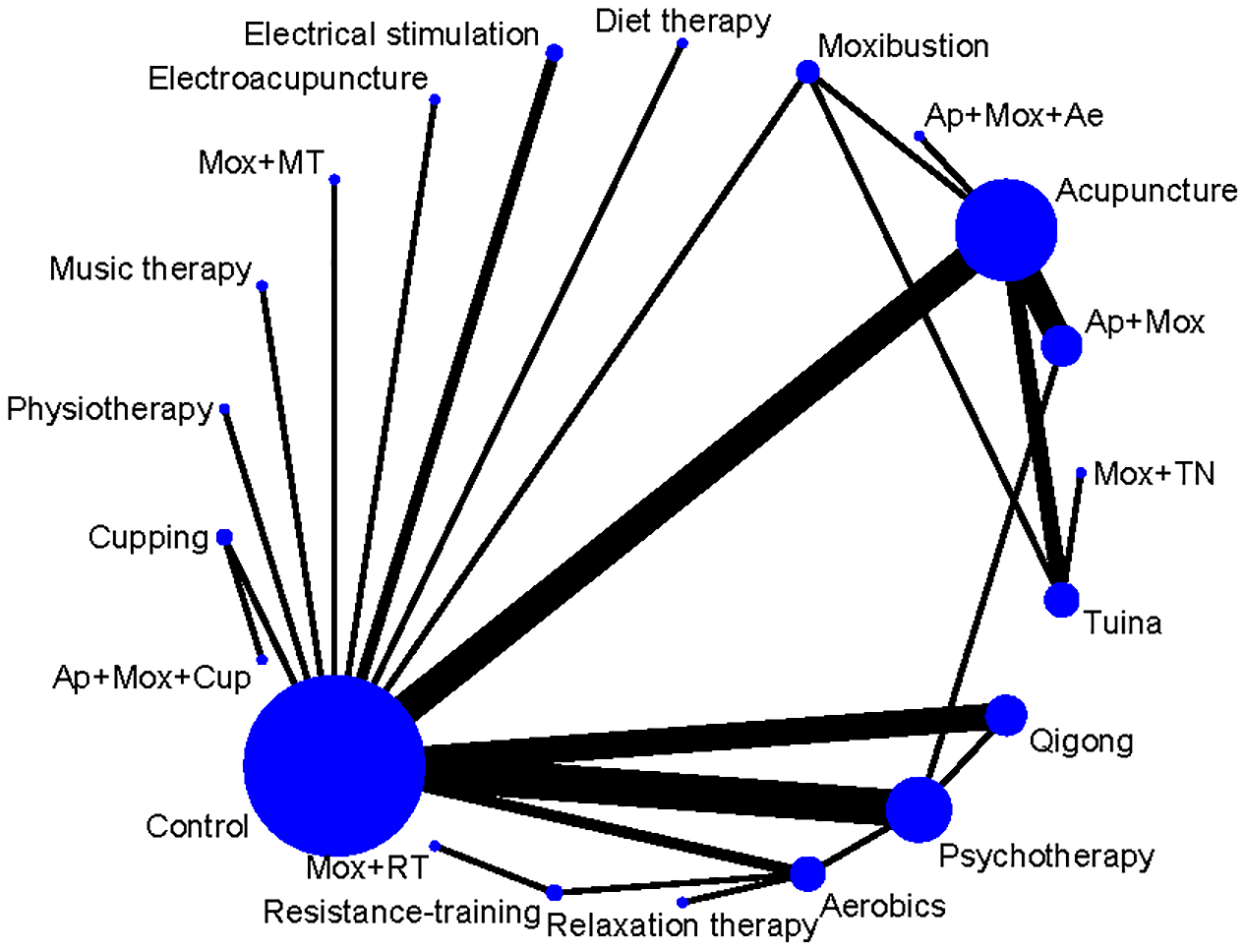
Network diagram of non-pharmacological therapies for treating depressive symptoms in patients with CFS. Ae, Acupoints embedding; AER, Aerobics; AP, Acupuncture; Cup, Cupping; Mox, Moxibustion; MT, Music therapy; Psy, Psychotherapy; RT, Resistance training.

In the network diagram, each dot symbolizes an intervention measure, and the size of the dot is positively correlated with the number of RCTs involved in each intervention measure; the larger the dot, the more studies are included. The lines connecting two dots indicate direct comparisons between these two interventions, and the thickness of the lines symbolizes the number of RCTs between the two treatment regimens; the thicker line indicates more comparative studies (**Figure 3**). The node splitting method was employed to analyze the results of each closed loop. P values of outcome measures were all greater than 0.05, suggesting no local inconsistency (**Supplementary Figure 1**). PSRF was equal to 1, indicating that the model was completely convergent. The specific convergence results are shown in **Supplementary Figures 2**, **3**.

#### 3.3.2 Summary results for each outcome measure

47 studies reported depression scale scores. Overall heterogeneity was 90%, indicating high heterogeneity (**Supplementary Figure 4**), so a random-effects model was selected. NMA results revealed that compared with the control group, CFS patients who received Ap, Mox, Tuina, diet therapy, electrical stimulation, Ap + Mox, or Ap + Mox + Ae had lowered depression scale scores (Control vs Ap: SMD = -1.91, 95% CI: -2.9 to -0.94; Control vs. Mox: SMD = -2.91, 95% CI: -4.61 to -1.22; Control vs. Tuina: SMD = -2, 95% CI: -3.64 to -0.38; Control vs Diet therapy: SMD = -5.64, 95% CI: -8.98 to -2.29; Electrical stimulation vs Control: SMD = 1.9, 95% CI: 0.03 to 3.78; Ap + Mox vs Control: SMD = 2.53, 95% CI: 1.17 to 3.91; Ap + Mox + Ae vs Control: SMD = 3.16, 95% CI: 0.39 to 5.98). Compared with the control group, the four exercise therapies (relaxation therapy, AER, RT, and Qigong) did not markedly improve depressive symptoms in CFS patients (Control vs. Relaxation therapy: SMD = 0.73, 95% CI: -1.23 to 2.71; Control vs. AER: SMD = -0.38, 95% CI: -1.81 to 1.05; Control vs. RT: SMD = -0.73, 95% CI: -3.8 to 2.33; Control vs. Qigong: SMD = -0.43, 95% CI: -1.52 to 0.66).

Compared with Psy, CFS patients who received Ap and Mox treatment showed notable decreases in depression scale scores (Psy vs Ap: SMD = -1.37, 95% CI: -2.64 to -0.11; Psy vs Mox: SMD = -2.37, 95% CI: -4.27 to -0.48).

Compared with AER, CFS patients who received Mox, diet therapy, or Ap + Mox treatment showed significant reductions in depression scale scores (Mox vs AER: SMD = -2.54, 95% CI: -4.75 to -0.31; AER vs. diet therapy: SMD = 5.26, 95% CI: 1.61 to 8.9; AER vs. Ap + Mox: SMD = 2.15, 95% CI: 0.2 to 4.12) (**Table 2**).

**Table 2:** Pairwise comparisons of the effect of non-exercise therapies on the reduction of depression scale scores in CFS patients.

Interventions/SMD (95% Crl)																				
Acupuncture																				
1 (-0.62, 2.61)	**Moxibustion**																			
-0.74 (-3.55, 2.04)	-1.75 (-4.86, 1.38)	**Cupping**																		
0.09 (-1.29, 1.46)	-0.92 (-2.68, 0.88)	0.83 (-2.24, 3.92)	**Tuina**																	
-1.25 (-4.06, 1.52)	-2.26 (-5.39, 0.84)	-0.51 (-4.21, 3.21)	-1.34 (-4.44, 1.72)	**Music therapy**																
-0.63 (-3.44, 2.18)	-1.62 (-4.77, 1.51)	0.12 (-3.6, 3.84)	-0.71 (-3.82, 2.39)	0.63 (-3.09, 4.35)	**Electroacupuncture**															
**-2.64 (-4.85, -0.46)**	**-3.64 (-6.25, -1.04)**	-1.9 (-5.16, 1.38)	**-2.73 (-5.3, -0.18)**	-1.39 (-4.67, 1.89)	-2.02 (-5.33, 1.28)	**Relaxation therapy**														
**-1.37 (-2.64, -0.11)**	**-2.37 (-4.27, -0.48)**	-0.63 (-3.4, 2.15)	-1.46 (-3.29, 0.36)	-0.12 (-2.88, 2.67)	-0.74 (-3.53, 2.05)	1.27 (-0.84, 3.4)	**Psychotherapy**													
-1.54 (-3.26, 0.18)	**-2.54 (-4.75, -0.31)**	-0.79 (-3.76, 2.2)	-1.62 (-3.79, 0.54)	-0.28 (-3.26, 2.71)	-0.91 (-3.91, 2.09)	1.11 (-0.87, 3.09)	-0.16 (-1.73, 1.4)	**Aerobics**												
**3.73 (0.23, 7.2)**	2.72 (-1.03, 6.48)	**4.47 (0.23, 8.71)**	3.64 (-0.09, 7.35)	**4.98 (0.74, 9.23)**	**4.35 (0.09, 8.61)**	**6.37 (2.49, 10.25)**	**5.1 (1.63, 8.55)**	**5.26 (1.61, 8.9)**	**Diet therapy**											
-1.19 (-4.4, 2.01)	-2.19 (-5.69, 1.3)	-0.44 (-4.46, 3.58)	-1.27 (-4.73, 2.16)	0.07 (-3.98, 4.11)	-0.56 (-4.61, 3.47)	1.46 (-1.89, 4.81)	0.19 (-2.94, 3.3)	0.35 (-2.35, 3.05)	**-4.91 (-9.44, -0.37)**	**Resistance-training**										
**-1.48 (-2.95, -0.04)**	**-2.49 (-4.5, -0.46)**	-0.74 (-3.57, 2.09)	-1.57 (-3.54, 0.37)	-0.23 (-3.06, 2.61)	-0.86 (-3.72, 2)	1.16 (-1.09, 3.4)	-0.11 (-1.43, 1.2)	0.05 (-1.74, 1.83)	**-5.21 (-8.73, -1.7)**	-0.3 (-3.54, 2.94)	**Qigong**									
**-1.91 (-2.9, -0.94)**	**-2.91 (-4.61, -1.22)**	-1.17 (-3.78, 1.45)	**-2 (-3.64, -0.38)**	-0.66 (-3.27, 1.96)	-1.29 (-3.92, 1.35)	0.73 (-1.23, 2.71)	-0.54 (-1.46, 0.37)	-0.38 (-1.81, 1.05)	**-5.64 (-8.98, -2.29)**	-0.73 (-3.8, 2.33)	-0.43 (-1.52, 0.66)	**Control**								
-0.01 (-2.13, 2.1)	-1.01 (-3.54, 1.52)	0.73 (-2.48, 3.96)	-0.09 (-2.59, 2.38)	1.25 (-1.97, 4.47)	0.62 (-2.63, 3.86)	2.64 (-0.1, 5.36)	1.36 (-0.72, 3.45)	1.52 (-0.82, 3.89)	-3.73 (-7.56, 0.09)	1.18 (-2.4, 4.76)	1.48 (-0.69, 3.65)	**1.9 (0.03, 3.78)**	**Electrical stimulation**							
-2.08 (-4.93, 0.77)	-3.08 (-6.25, 0.09)	-1.34 (-5.08, 2.41)	-2.16 (-5.31, 0.96)	-0.82 (-4.57, 2.94)	-1.46 (-5.19, 2.31)	0.56 (-2.75, 3.9)	-0.71 (-3.54, 2.13)	-0.55 (-3.57, 2.5)	**-5.8 (-10.07, -1.51)**	-0.89 (-4.94, 3.19)	-0.59 (-3.46, 2.3)	-0.17 (-2.84, 2.51)	-2.07 (-5.34, 1.19)	**Physiotherapy**						
0.61 (-0.48, 1.71)	-0.38 (-2.31, 1.55)	1.36 (-1.59, 4.32)	0.53 (-1.21, 2.27)	1.87 (-1.07, 4.83)	1.24 (-1.72, 4.22)	**3.26 (0.88, 5.67)**	**1.99 (0.49, 3.5)**	**2.15 (0.2, 4.12)**	-3.11 (-6.71, 0.51)	1.8 (-1.52, 5.15)	**2.1 (0.38, 3.83)**	**2.53 (1.17, 3.91)**	0.62 (-1.69, 2.95)	2.69 (-0.31, 5.7)	**Ap+Mox**					
1.25 (-1.37, 3.88)	0.25 (-2.83, 3.35)	1.99 (-1.83, 5.85)	1.16 (-1.8, 4.14)	2.5 (-1.3, 6.35)	1.88 (-1.97, 5.75)	**3.89 (0.48, 7.33)**	2.62 (-0.27, 5.55)	2.78 (-0.35, 5.94)	-2.48 (-6.83, 1.89)	2.44 (-1.68, 6.58)	2.73 (-0.24, 5.74)	**3.16 (0.39, 5.98)**	1.26 (-2.1, 4.64)	3.32 (-0.53, 7.21)	0.63 (-2.21, 3.48)	**Ap+Mox+Ae**				
-0.18 (-3.13, 2.79)	-1.18 (-4.34, 2)	0.57 (-3.47, 4.63)	-0.26 (-2.87, 2.37)	1.08 (-2.96, 5.12)	0.45 (-3.6, 4.53)	2.47 (-1.19, 6.14)	1.2 (-1.99, 4.39)	1.36 (-2.03, 4.75)	-3.89 (-8.45, 0.65)	1.01 (-3.33, 5.36)	1.31 (-1.95, 4.6)	1.74 (-1.34, 4.84)	-0.16 (-3.77, 3.45)	1.9 (-2.17, 5.99)	-0.79 (-3.93, 2.35)	-1.42 (-5.4, 2.54)	**Mox+TN**			
-0.46 (-4.59, 3.68)	-1.46 (-5.8, 2.9)	0.28 (-4.49, 5.09)	-0.55 (-4.87, 3.78)	0.8 (-4, 5.58)	0.17 (-4.63, 4.97)	2.18 (-2.06, 6.44)	0.91 (-3.13, 4.99)	1.07 (-2.66, 4.83)	-4.18 (-9.41, 1.04)	0.72 (-1.85, 3.33)	1.02 (-3.12, 5.19)	1.46 (-2.57, 5.48)	-0.44 (-4.89, 4.01)	1.63 (-3.23, 6.45)	-1.07 (-5.3, 3.17)	-1.71 (-6.61, 3.18)	-0.29 (-5.33, 4.78)	**Mox+RT**		
0.38 (-2.44, 3.19)	-0.62 (-3.76, 2.52)	1.13 (-2.61, 4.86)	0.29 (-2.83, 3.38)	1.64 (-2.09, 5.37)	1 (-2.72, 4.73)	3.03 (-0.27, 6.31)	1.76 (-1.05, 4.55)	1.92 (-1.09, 4.91)	-3.33 (-7.59, 0.9)	1.57 (-2.47, 5.58)	1.87 (-0.99, 4.72)	2.3 (-0.34, 4.93)	0.39 (-2.85, 3.62)	2.46 (-1.31, 6.21)	-0.23 (-3.21, 2.73)	-0.86 (-4.72, 2.96)	0.55 (-3.51, 4.59)	0.84 (-3.97, 5.62)	**Mox+MT**	
0.29 (-3.55, 4.11)	-0.71 (-4.77, 3.35)	1.03 (-1.56, 3.64)	0.2 (-3.84, 4.25)	1.55 (-2.99, 6.08)	0.92 (-3.63, 5.45)	2.93 (-1.26, 7.12)	1.66 (-2.16, 5.47)	1.82 (-2.13, 5.78)	-3.44 (-8.42, 1.55)	1.47 (-3.31, 6.29)	1.77 (-2.06, 5.61)	2.2 (-1.5, 5.9)	0.3 (-3.84, 4.43)	2.37 (-2.21, 6.93)	-0.32 (-4.29, 3.61)	-0.96 (-5.61, 3.66)	0.47 (-4.36, 5.25)	0.75 (-4.7, 6.19)	-0.09 (-4.65, 4.46)	**Ap+Mox+Cup**

The table presents Standardized Mean Differences (SMDs) with 95% credible intervals (95CrIs) comparing column interventions versus row interventions. A smaller SMD indicates greater efficacy in reducing depression scores, while an SMD of 1 denotes no difference between interventions. Bolded values indicate statistically significant improvements in depression score reduction. Abbreviations: Ae, Acupoints embedding; AER, Aerobics; AP, Acupuncture; Cup, Cupping; Mox, Moxibustion; MT, Music therapy; Psy, Psychotherapy; RT, Resistance training.

**Figure 4:**
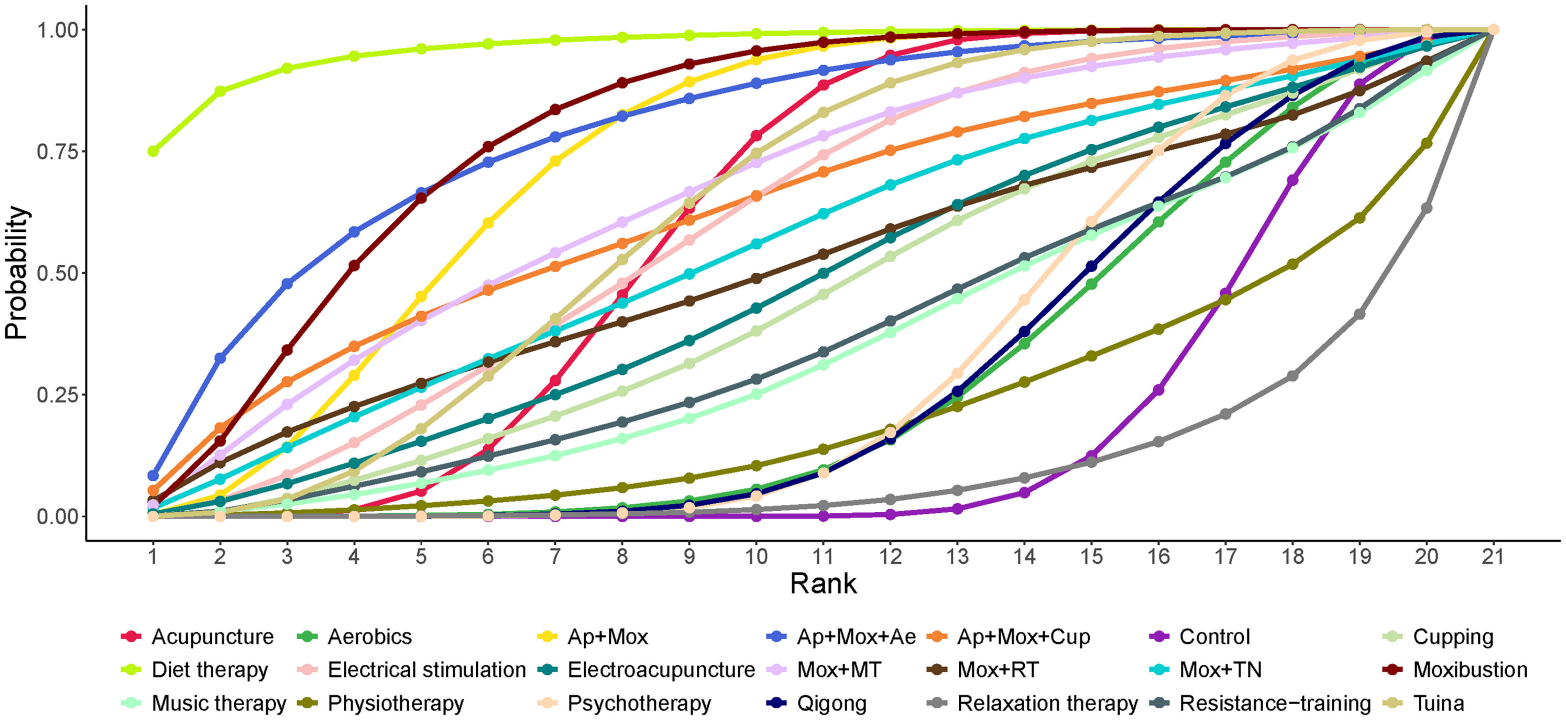
Area under the SUCRA curve for the effect of different non-pharmacological therapies on reducing depression scale scores in patients with CFS. Ae, Acupoints embedding; AER, Aerobics; AP, Acupuncture; Cup, Cupping; Mox, Moxibustion; MT, Music therapy; Psy, Psychotherapy; RT, Resistance training.

SUCRA probability ranking results showed diet therapy (96.72%) > Mox (79.99%) > Ap + Mox + Ae (79.59%) > Ap + Mox (74.27%) > Mox + MT (66.38%). Diet therapy alone had the greatest effect on reducing depression scores in CFS patients (**Figure 4**).

### 3.4 Publication bias

**Figure 5:**
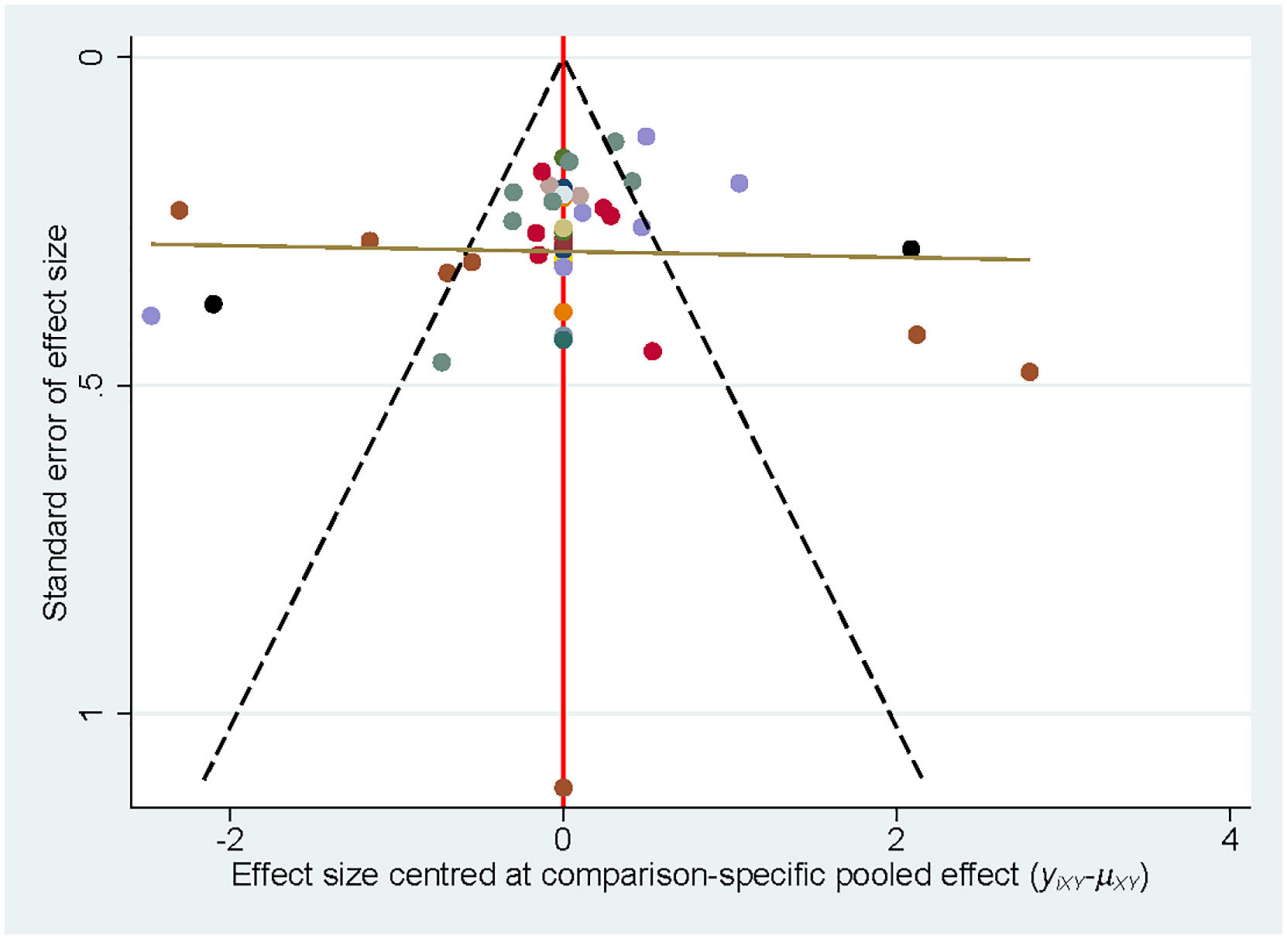
Funnel plot of depression scales in patients with CFS.

Publication bias was examined by plotting a correction-comparison funnel plot. The results showed symmetrical funnel plots, indicating no publication bias (**Figure 5**).

### 3.5 Meta-regression

A meta-regression analysis was performed on the sample size to explore the possible sources of heterogeneity and the stability of results. The results showed that the regression results were significant in pairwise comparisons of some intervention methods, indicating that sample size is a possible source of heterogeneity.

## 4 Discussion

In our results, Ap, Mox, Tuina, diet therapy, electrical stimulation, Ap + Mox, and Ap + Mox + Ae significantly reduced depression scores in CFS patients compared to the control intervention, demonstrating reliable efficacy in treating depression.

The high comorbidity rate between CFS and depression depends on the shared pathological mechanisms between the two conditions. Nakatomi et al. (29) found that high expression of activated microglia translocator protein in the hippocampus of CFS patients was positively correlated with depression scores. CFS patients with comorbid major depression exhibit mixed immune responses and neuroinflammation in widespread brain regions, which is associated with the severity of neuropsychological symptoms (30). CFS patients exhibit reduced tricarboxylic acid cycle activity, mitochondrial energy metabolism dysfunction, and systemic metabolic dysfunction (31). Mitochondrial energy metabolism dysfunction is also a pathological mechanism of depression (32). CFS and depression patients show abnormalities in hypothalamic-pituitary-adrenal (HPA) axis regulation and disrupted mechanisms of cortisol secretion feedback (33). Depressed and physically inactive CFS patients may present with hypocortisolemia (34). In CFS patients, the binding potential values of central 5-HT1A receptors, as well as the levels of serotonin (5-HT) transporters and receptors, are significantly reduced (35, 36). Meanwhile, 5-HT dysfunction is already recognized as a contributing factor to depression (37). Additionally, both CFS and depression patients exhibit a significant reduction in gray matter volume in the hippocampus and prefrontal cortex (38, 39). CFS patients exhibit reduced heart rate variability and cerebral blood flow (CBF) (40). A decrease in whole-brain average CBF is notably associated with experiencing more depressive episodes in adulthood (41). In summary, CFS and depression share mechanistic changes in immune-inflammatory responses, neuroendocrine regulation, and brain structure and function. These changes explain the high comorbidity rate between the two conditions and provide potential therapeutic targets for treating depressive symptoms in CFS patients.

In our results, diet therapy ranked first in terms of efficacy. The intervention measure in this study was chocolate rich in polyphenols. The active ingredient in cocoa, polyphenols, is a common antioxidant in food. Research has shown that the flavonoids in cocoa polyphenols have protective effects on neurons, shielding them from damage caused by oxidative stress (42). In addition, polyphenols regulate the transmission of monoamine neurotransmitters, reduce the circulation and brain concentration of pro-inflammatory mediators, regulate the HPA axis, promote hippocampal neurogenesis, enhance brain-derived neurotrophic factor (BDNF), and show antidepressant effects by regulating the composition of the intestinal microbiota via supporting the growth of beneficial bacteria and inhibiting pathogenic bacteria (43, 44). Polyphenols have also shown positive results in alleviating CFS symptoms (45). The study we included excluded the sweetness of chocolate and the potential pleasure derived from its energy-boosting effects. The study had high quality and low risk of bias. However, the sample size was small, and only one study was included. Therefore, the best efficacy of diet therapy (high-cocoa liquid/polyphenol-rich chocolate) in improving depressive symptoms in CFS patients should be interpreted with caution. Further studies are warranted to substantiate the efficacy of cocoa or diet therapy.

Ap, Mox, and Ap + Mox also showed favorable therapeutic effects. Ap and Mox are widely used traditional Chinese medicine methods. Both can dredge the meridians, promote the movement of qi and blood, and modulate the balance of qi and blood. Mox has a warming effect and is widely applied for diseases with weak symptoms, including CFS, which may explain why Mox is more effective than Ap. Laboratory evidence suggests that Mox effectively regulates the behavior, immune function, and HPA axis of rats with CFS, thereby alleviating fatigue symptoms (46, 47). Mox can also repair the intestinal barrier by regulating the intestinal flora structure of patients, significantly improving the fatigue status of CFS patients (48). Ap can suppress glial cell activation, mitigate neuroinflammation (49), repair damaged neural tissue structure in the hippocampus (50), regulate HPA axis dysfunction, inhibit HPA axis hyperactivity (51), and adjust gut microbiota abundance, thereby alleviating depressive symptoms in CFS.

Tuina therapy is a type of external treatment in traditional Chinese medicine, which has the functions of dredging meridians, regulating internal organs, and relaxing the mind and body. It also has the advantages of being simple, safe, and having no adverse reactions (52). Abdominal massage can reduce the organ indices of the hypothalamus, pituitary gland, and adrenal glands, key organs in the HPA axis in CFS rats, alleviate damage to hippocampal tissue, inhibit hippocampal cell apoptosis, and increase hippocampal cell viability, thereby helping to maintain the normal physiological functions of hippocampal neurons (53). In addition, in improving depressive behavior in rats with chronic stress, it can upregulate ERK phosphorylation in the hippocampus and prefrontal cortex, activate the ERK pathway, and promote the expression of the effector protein BDNF (54).

Electrical stimulation therapy includes tDCS and transcutaneous electrical nerve stimulation (TENS), which use weak electrical stimulation to regulate neural activity or relieve pain. Transcranial electrical stimulation is divided into anodal (positive) and cathodal (negative). Positive stimulation enhances cortical excitability, while negative stimulation reduces cortical excitability, primarily regulating brain neuronal activity and widely applied in the field of psychiatry. TEAS, on the other hand, acts on the skin surface. TENS can enhance the learning and memory abilities of CFS rats, possibly by improving tissue structure of the hippocampal CA1 region and upregulating ERK/CREB/BDNF expression (55). TENS can reduce serum IL-1β and IL-6 concentrations in patients with late-pregnancy depression (56). Through functional magnetic resonance imaging, Ma et al. (57) discovered that transcutaneous cranial-auricular acupoint electrical stimulation can modulate the function of the abnormal emotion-related brain network ‘insula-frontal lobe-limbic system’ and exert an antidepressant effect. Research has found that patients with depression exhibit reduced CBF and slowed metabolism in the left dorsolateral prefrontal cortex (DLPFC), while the right DLPFC shows accelerated metabolism. tDCS can enhance the excitability of the left DLPFC while inhibiting the right DLPFC, thereby regulating the activity of the brain’s emotional circuitry and alleviating depressive symptoms by stimulating the prefrontal cortex (58). A systematic review on fibromyalgia suggests that when tDCS is applied to the DLPFC, it improves patients’ fatigue symptoms (59).

Additionally, our results suggested that four types of exercise therapy, relaxation therapy, AER, RT, and Qigong, were less effective. P. Windthorst (60) et al. failed to observe any significant therapeutic effects of graded exercise therapy in improving depressive symptoms in CFS patients. Graded exercise therapy may exacerbate symptoms in certain CFS patients, possibly related to the characteristic PEM of CFS (61). Psychotherapy also did not show significant effects, which may be related to several factors, including the fact that CBT was not designed to target depressive symptoms in the included studies and the diverse etiology of CFS.

According to the SUCRA probability ranking results, we should give priority to recommending diet therapy as an intervention for depressive symptoms in CFS patients. However, due to the limited number of articles included in the diet therapy intervention, its therapeutic effect may be exaggerated. Therefore, we are currently more inclined to recommend Mox, which ranks second or third in the SUCRA probability ranking, and the combined treatment of Ap + Mox + Ae.

To our knowledge, this is the first study comparing non-pharmacological therapies for depression or depressive symptoms in patients with CFS. We conducted an extensive literature review and included the most comprehensive studies to date on non-pharmacological therapies for treating depressive symptoms in CFS. These strengths enable our findings to support clinicians in selecting appropriate treatment options based on patient tolerance, thereby facilitating personalized treatment approaches. Additionally, our findings provide evidence for the development of clinical guidelines.

However, this study has some limitations. The included studies exhibit a certain degree of heterogeneity. First, the scales used for depression outcomes were inconsistent. Although there was no significant heterogeneity in the outcomes when the SMD was used to merge the effect size, it could only reflect the aggravation or relief of the patient’s depressive symptoms through numerical changes, and there were still limitations in the interpretation of the results. The risk of bias in most included studies was medium; some interventions were included in a few studies with small sample sizes, so the results should be viewed with caution. Clinical studies with large sample sizes should be supplemented in future studies to reduce heterogeneity. Therefore, we recommend that future high-quality RCTs focus more on non-pharmacological therapies for treating depressive symptoms in CFS patients to enhance the reliability of research results. These results strengthen the existing evidence and provide valuable insights for patients, healthcare providers, and policymakers.

## 5 Conclusion

This study evaluated the efficacy of various non-pharmacological therapies in alleviating depression or depressive symptoms in patients with CFS through network analysis. The results showed that Ap, Mox, Tuina, diet therapy, electrical stimulation, Ap + Mox, and Ap + Mox + Ae were significantly more effective than the control intervention in reducing depression scale scores in CFS patients, demonstrating reliable efficacy in antidepressant effects. Diet therapy was the most effective, followed by moxibustion, Ap + Mox + Ae, Ap + Mox, and Mox + MT. Further high-quality RCTs are warranted to enrich this field of research.

## Data Availability

The original contributions presented in the study are included in the article/**Supplementary Material**. Further inquiries can be directed to the corresponding author.

## Supplementary material

Supplementary Figure 1 Local inconsistency test for non-pharmacological therapies for depressive symptoms in CFS patients.

Supplementary Figure 2 Density plot for convergence results.

Supplementary Figure 3 Trajectory plot for PSRF convergence.

Supplementary Figure 4 Local heterogeneity test results.
