# Supplementary figures and images for: Effect of nonpharmacologic therapies on depressive symptoms in patients with chronic fatigue syndrome: a network meta-analysis
Source: Front Psychiatry. 2025 Aug 19;16:1657615. doi: 10.3389/fpsyt.2025.1657615 (PMC12401970; doi:10.3389/fpsyt.2025.1657615)

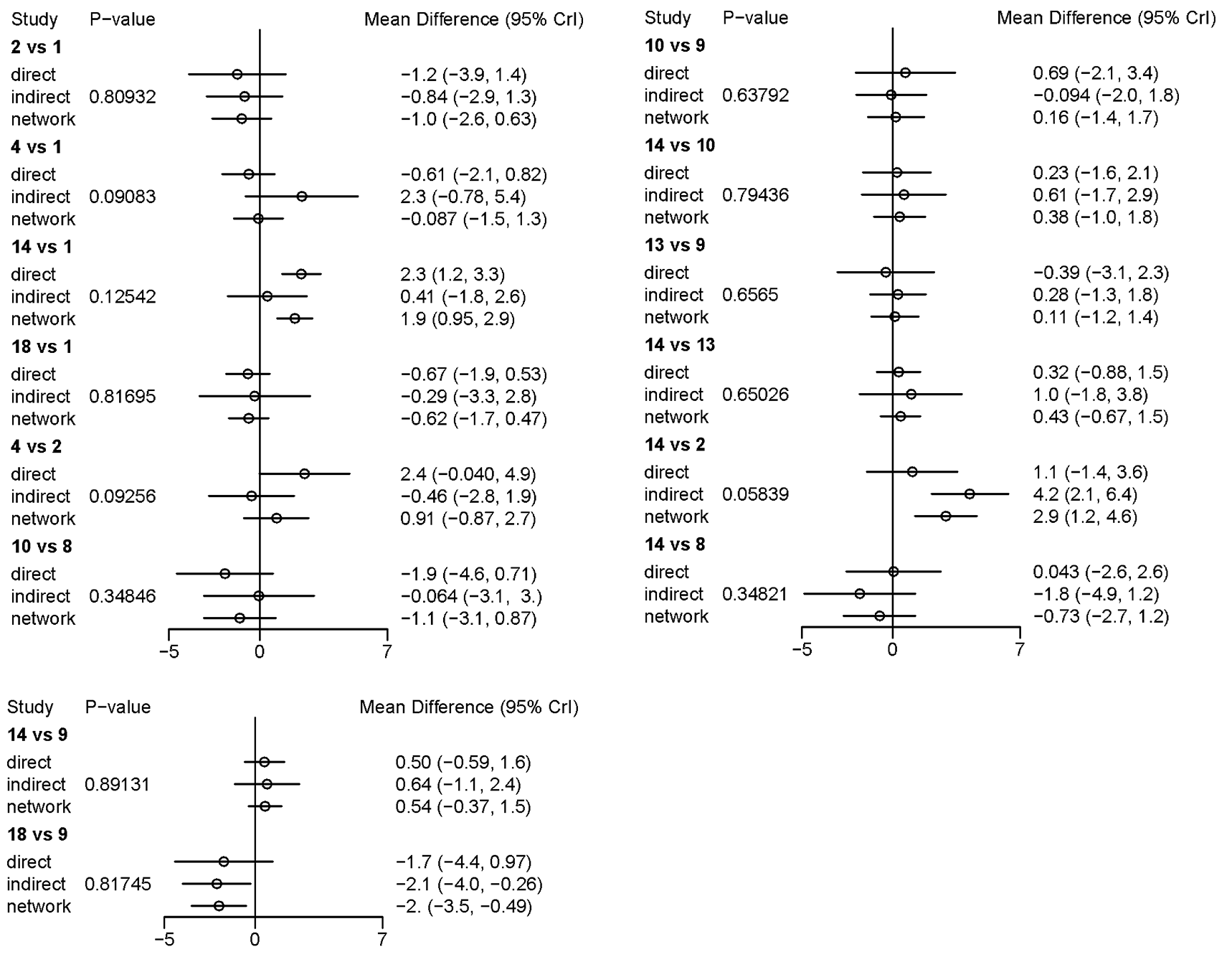

Supplement: Supplementary Figure 1 — Local inconsistency test for non-pharmacological therapies for depressive symptoms in CFS patients. [file Image1.tif]

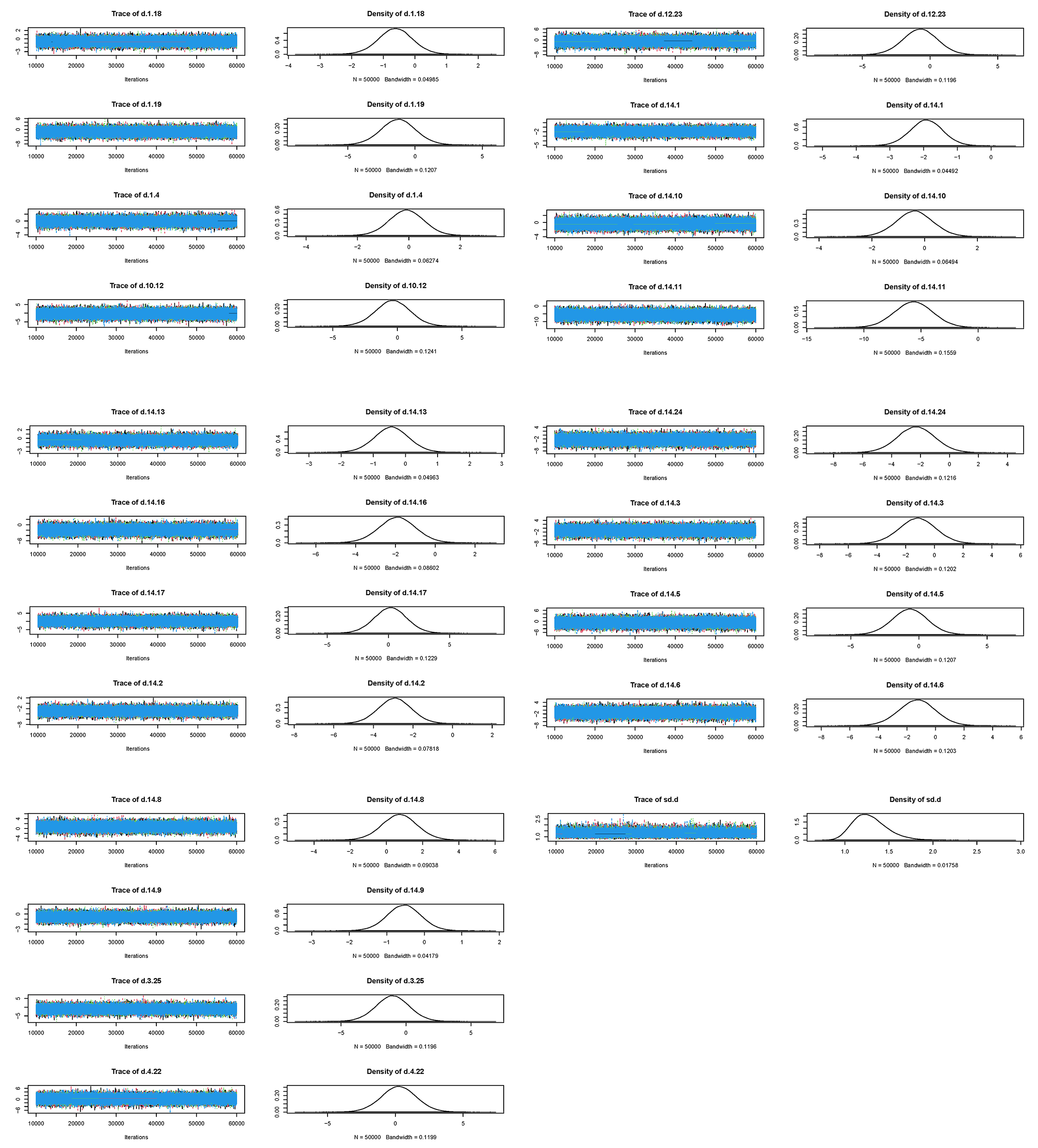

Supplement: Supplementary Figure 2 — Density plot for convergence results. [file Image2.tif]

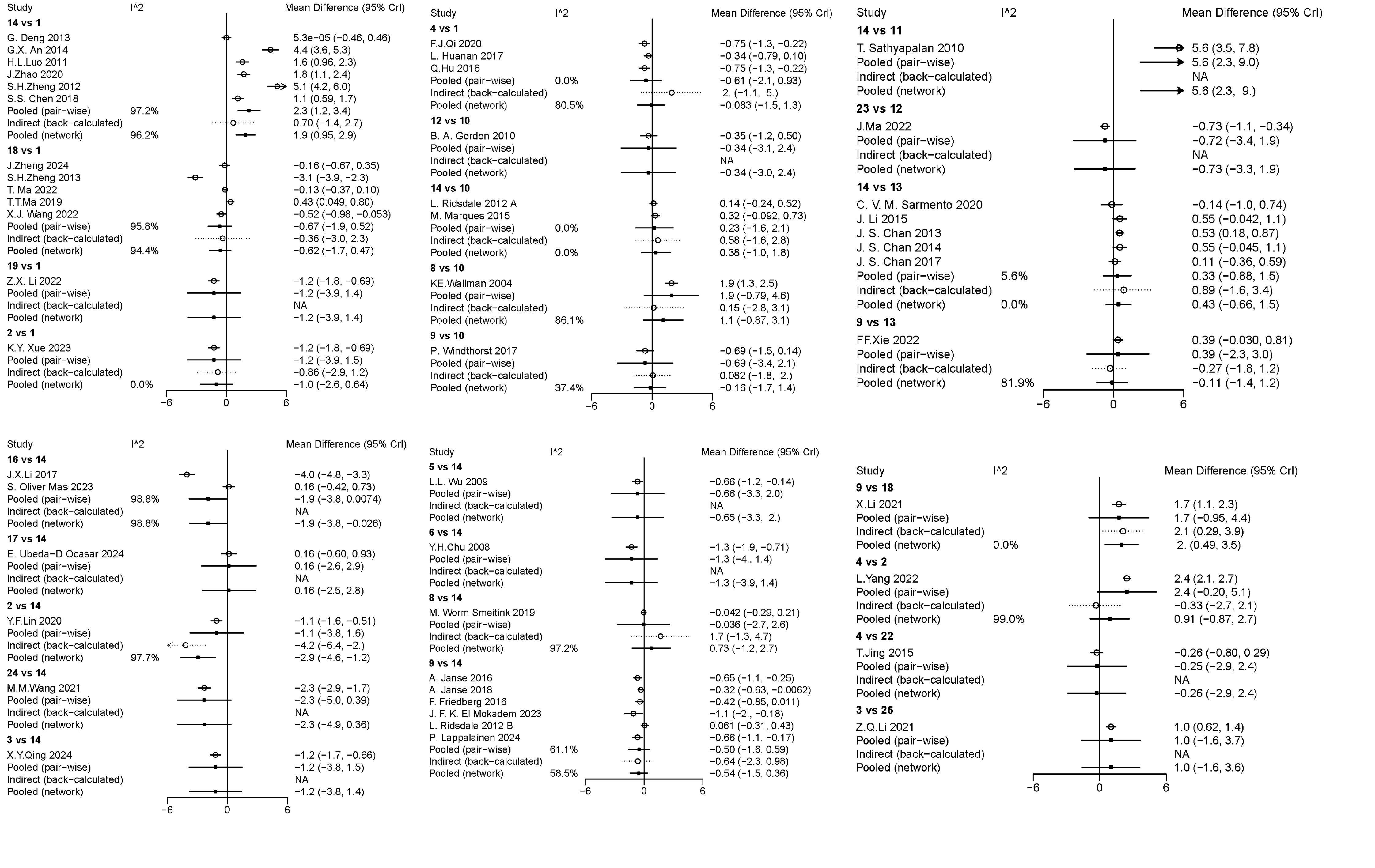

Supplement: Supplementary Figure 3 — Trajectory plot for PSRF convergence. [file Image3.tif]

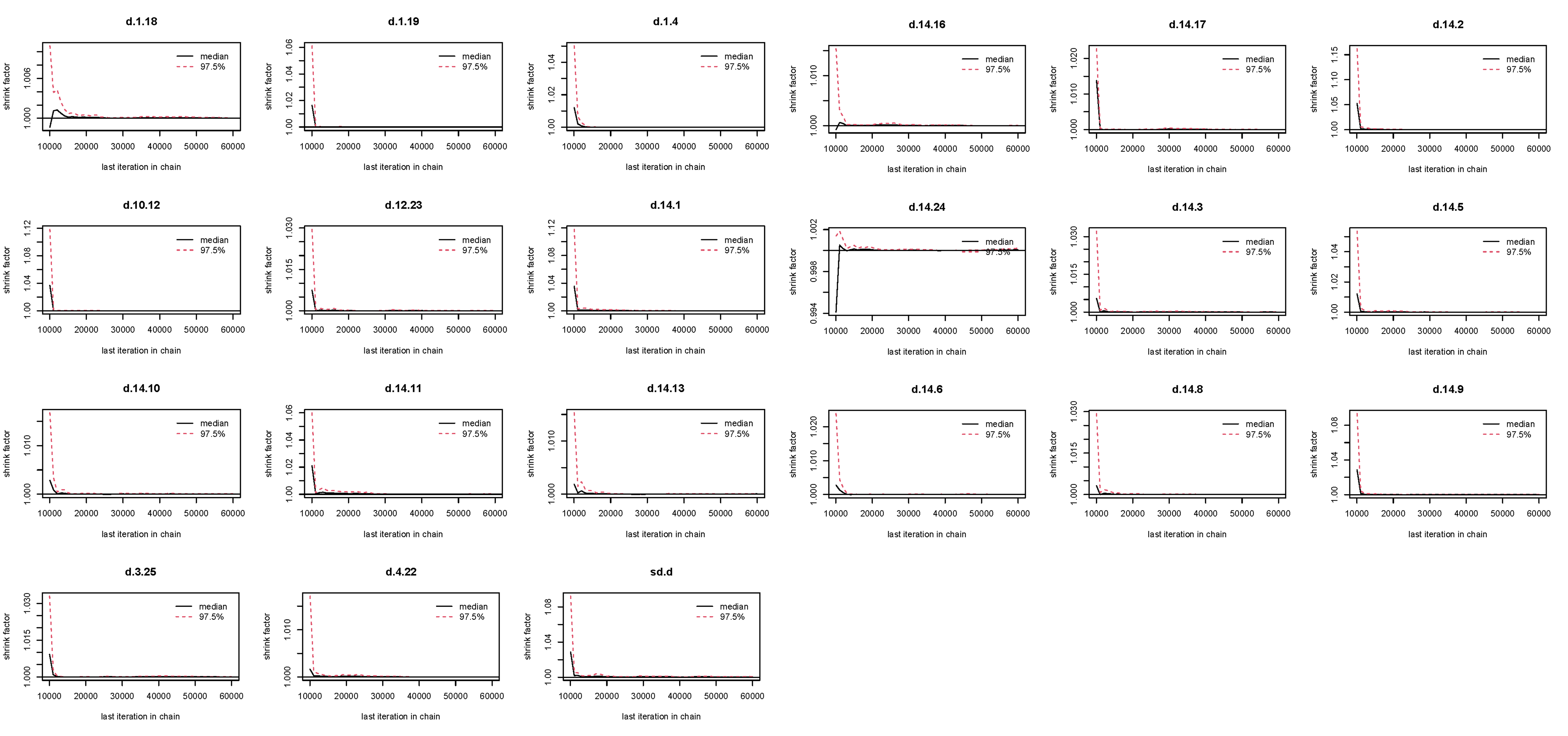

Supplement: Supplementary Figure 4 — Local heterogeneity test results. [file Image4.tif]
