# Supplementary material for: Effect of nonpharmacologic therapies on depressive symptoms in patients with chronic fatigue syndrome: a network meta-analysis
Source: Front Psychiatry. 2025 Aug 19;16:1657615. doi: 10.3389/fpsyt.2025.1657615 (PMC12401970; doi:10.3389/fpsyt.2025.1657615)
Supplement: Supplementary file 5 [file Table1.docx]

**Table S1.** **The search strategy (Pubmed)**

| Search number | Query | Results |
| --- | --- | --- |
| 1 | Fatigue Syndrome, Chronic[MeSH Terms] | 6502 |
| 2 | "Yuppie flu"[Title/Abstract] OR "Systemic Exertion Intolerance Disease"[Title/Abstract] OR "syndrome of chronic fatigue"[Title/Abstract] OR "Royal Free Disease"[Title/Abstract] OR "Postviral Fatigue Syndromes"[Title/Abstract] OR "Postviral Fatigue Syndrome"[Title/Abstract] OR "Myalgic Encephalomyelitis"[Title/Abstract] OR "Iceland disease"[Title/Abstract] OR "fatigue syndrome"[Title/Abstract] OR "epidemic neuromyasthenia"[Title/Abstract] OR "Chronic Fatigue Syndrome*"[Title/Abstract] OR "Chronic Fatigue Fibromyalgia Syndrome"[Title/Abstract] OR "Chronic Fatigue Disorder*"[Title/Abstract] OR "Chronic Fatigue and Immune Dysfunction Syndrome"[Title/Abstract] OR "Chronic Fatigue Fibromyalgia Syndromes"[Title/Abstract] OR "Chronic Fatigue Fibromyalgia Syndrome"[Title/Abstract] OR "chronic fatigue syndrome"[Title/Abstract] OR "chronic fatigue and immune dysfunction syndrome"[Title/Abstract] OR "chronic fatigue"[Title/Abstract] OR "benign myalgic encephalomyelitis"[Title/Abstract] OR "Akureyri disease"[Title/Abstract] | 9789 |
| 3 | (Depression[MeSH Terms]) OR (Depressive Disorder[MeSH Terms]) | 277996 |
| 4 | "central depression"[Title/Abstract] OR "clinical depression"[Title/Abstract] OR "Depression"[Title/Abstract] OR "depressive disease"[Title/Abstract] OR "Depressive Disorder"[Title/Abstract] OR "Depressive Disorders"[Title/Abstract] OR "depressive episode"[Title/Abstract] OR "depressive illness"[Title/Abstract] OR "Depressive Neuroses"[Title/Abstract] OR "Depressive Neurosis"[Title/Abstract] OR "depressive personality disorder"[Title/Abstract] OR "depressive state"[Title/Abstract] OR "Depressive Symptom*"[Title/Abstract] OR "Depressive Syndrome*"[Title/Abstract] OR "depressivity"[Title/Abstract] OR "Emotional Depression"[Title/Abstract] OR "Endogenous Depression*"[Title/Abstract] OR "Melancholia"[Title/Abstract] OR "Melancholias"[Title/Abstract] OR "mental depression"[Title/Abstract] OR "Neurotic Depression*"[Title/Abstract] OR "parental depression"[Title/Abstract] OR "Unipolar Depression*"[Title/Abstract] | 524905 |
| 5 | "randomised controlled study"[Title/Abstract] OR "randomised controlled trial"[Title/Abstract] OR "randomized controlled study"[Title/Abstract] OR "randomized controlled trial"[Title/Abstract] OR "random*"[Title/Abstract] OR "trial, randomized controlled"[Title/Abstract] OR "Clinical Trials, Randomized"[Title/Abstract] OR "Trials, Randomized Clinical"[Title/Abstract] | 1614984 |
| 6 | (1 OR 2) AND (3 OR 4) AND 5 | 221 |

**The search strategy (Embase)**

| Search Number | Query | Results |
| --- | --- | --- |
| 1 | 'chronic fatigue syndrome'/exp | 23263 |
| 2 | 'yuppie flu':ab,ti,kw OR 'systemic exertion intolerance disease':ab,ti,kw OR 'syndrome of chronic fatigue':ab,ti,kw OR 'royal free disease':ab,ti,kw OR 'postviral fatigue syndromes':ab,ti,kw OR 'postviral fatigue syndrome':ab,ti,kw OR 'myalgic encephalomyelitis':ab,ti,kw OR 'iceland disease':ab,ti,kw OR 'fatigue syndrome':ab,ti,kw OR 'epidemic neuromyasthenia':ab,ti,kw OR 'chronic fatigue syndrome*':ab,ti,kw OR 'chronic fatigue disorder*':ab,ti,kw OR 'chronic fatigue fibromyalgia syndromes':ab,ti,kw OR 'chronic fatigue fibromyalgia syndrome':ab,ti,kw OR 'chronic fatigue syndrome':ab,ti,kw OR 'chronic fatigue and immune dysfunction syndrome':ab,ti,kw OR 'chronic fatigue':ab,ti,kw OR 'benign myalgic encephalomyelitis':ab,ti,kw OR 'akureyri disease':ab,ti,kw | 13095 |
| 3 | 'depression'/exp | 728551 |
| 4 | 'central depression':ab,ti,kw OR 'clinical depression':ab,ti,kw OR 'depression':ab,ti,kw OR 'depressive disease':ab,ti,kw OR 'depressive disorder':ab,ti,kw OR 'depressive disorders':ab,ti,kw OR 'depressive episode':ab,ti,kw OR 'depressive illness':ab,ti,kw OR 'depressive neuroses':ab,ti,kw OR 'depressive neurosis':ab,ti,kw OR 'depressive personality disorder':ab,ti,kw OR 'depressive state':ab,ti,kw OR 'depressive symptom*':ab,ti,kw OR 'depressive syndrome*':ab,ti,kw OR 'depressivity':ab,ti,kw OR 'emotional depression':ab,ti,kw OR 'endogenous depression*':ab,ti,kw OR 'melancholia':ab,ti,kw OR 'melancholias':ab,ti,kw OR 'mental depression':ab,ti,kw OR 'neurotic depression*':ab,ti,kw OR 'parental depression':ab,ti,kw OR 'unipolar depression*':ab,ti,kw | 720239 |
| 5 | 'randomised controlled study':ab,ti,kw OR 'randomised controlled trial':ab,ti,kw OR 'randomized controlled study':ab,ti,kw OR 'randomized controlled trial':ab,ti,kw OR 'random*':ab,ti,kw OR 'trial, randomized controlled':ab,ti,kw OR 'clinical trials, randomized':ab,ti,kw OR 'trials, randomized clinical':ab,ti,kw | 2195019 |
| 6 | (1 OR 2) AND (3 OR 4) AND 5 | 519 |

**The search strategy (Cochrane)**

| Search Number | Query | Results |
| --- | --- | --- |
| 1 | MeSH descriptor: [Fatigue Syndrome, Chronic] explode all trees | 558 |
| 2 | (‘Yuppie flu’ OR ‘Systemic Exertion Intolerance Disease’ OR ‘syndrome of chronic fatigue’ OR ‘Royal Free Disease’ OR ‘Postviral Fatigue Syndromes’ OR ‘Postviral Fatigue Syndrome’ OR ‘Myalgic Encephalomyelitis’ OR ‘Iceland disease’ OR ‘fatigue syndrome’ OR ‘epidemic neuromyasthenia’ OR ‘Chronic Fatigue Syndrome*’ OR ‘Chronic Fatigue Fibromyalgia Syndrome’ OR ‘Chronic Fatigue Disorder*’ OR ‘Chronic Fatigue and Immune Dysfunction Syndrome’ OR ‘Chronic Fatigue Fibromyalgia Syndromes’ OR ‘Chronic Fatigue Fibromyalgia Syndrome’ OR ‘chronic fatigue syndrome’ OR ‘chronic fatigue and immune dysfunction syndrome’ OR ‘chronic fatigue’ OR ‘benign myalgic encephalomyelitis’ OR ‘Akureyri disease’):ab,ti,kw | 13472 |
| 3 | MeSH descriptor: [Depression] explode all trees | 18820 |
| 4 | MeSH descriptor: [Depressive Disorder] explode all trees | 16813 |
| 5 | (‘central depression’ OR ‘clinical depression’ OR ‘Depression’ OR ‘depressive disease’ OR ‘Depressive Disorder’ OR ‘Depressive Disorders’ OR ‘depressive episode’ OR ‘depressive illness’ OR ‘Depressive Neuroses’ OR ‘Depressive Neurosis’ OR ‘depressive personality disorder’ OR ‘depressive state’ OR ‘Depressive Symptom*’ OR ‘Depressive Syndrome*’ OR ‘depressivity’ OR ‘Emotional Depression’ OR ‘Endogenous Depression*’ OR ‘Melancholia’ OR ‘Melancholias’ OR ‘mental depression’ OR ‘Neurotic Depression*’ OR ‘parental depression’ OR ‘Unipolar Depression*’):ab,ti,kw | 116585 |
| 6 | (‘randomised controlled study’ OR ‘randomised controlled trial’ OR ‘randomized controlled study’ OR ‘randomized controlled trial’ OR ‘random*’ OR ‘trial, randomized controlled’ OR ‘Clinical Trials, Randomized’ OR ‘Trials, Randomized Clinical’):ab,ti,kw | 1375335 |
| 7 | (1 OR 2) AND (3 OR 4 OR 5) AND 6 | 2264 |

**The search strategy (Web Of Science)**

| Search Number | Query | Results |
| --- | --- | --- |
| 1 | "TS=((Yuppie flu) OR (Systemic Exertion Intolerance Disease) OR (syndrome of chronic fatigue) OR (Royal Free Disease) OR (Postviral Fatigue Syndromes) OR (Postviral Fatigue Syndrome) OR (Myalgic Encephalomyelitis) OR (Iceland disease) OR (fatigue syndrome) OR (epidemic neuromyasthenia) OR (Chronic Fatigue Syndrome*) OR (Chronic Fatigue Fibromyalgia Syndrome) OR (Chronic Fatigue Disorder*) OR (Chronic Fatigue and Immune Dysfunction Syndrome) OR (Chronic Fatigue Fibromyalgia Syndromes) OR (Chronic Fatigue Fibromyalgia Syndrome) OR (chronic fatigue syndrome) OR (chronic fatigue and immune dysfunction syndrome) OR (chronic fatigue) OR (benign myalgic encephalomyelitis) OR (Akureyri disease)) and Preprint Citation Index (Exclude – Database) " | 85768 |
| 2 | "TS=((central depression) OR (clinical depression) OR (Depression) OR (depressive disease) OR (Depressive Disorder) OR (Depressive Disorders) OR (depressive episode) OR (depressive illness) OR (Depressive Neuroses) OR (Depressive Neurosis) OR (depressive personality disorder) OR (depressive state) OR (Depressive Symptom*) OR (Depressive Syndrome*) OR (depressivity) OR (Emotional Depression) OR (Endogenous Depression*) OR (Melancholia) OR (Melancholias) OR (mental depression) OR (Neurotic Depression*) OR (parental depression) OR (Unipolar Depression*)) and Preprint Citation Index (Exclude – Database) " | 1334265 |
| 3 | "TS=((randomised controlled study) OR (randomised controlled trial) OR (randomized controlled study) OR (randomized controlled trial) OR (random*) OR (trial, randomized controlled) OR (Clinical Trials, Randomized) OR (Trials, Randomized Clinical)) and Preprint Citation Index (Exclude – Database) " | 4503180 |
| 4 | 1 AND 2 AND 3 and Preprint Citation Index (Exclude – Database) | 2766 |

**The search strategy (CNKI)**

| Search Number | Query | Results |
| --- | --- | --- |
| 1 | 慢性疲劳 + 慢性疲劳综合征 + '慢性疲劳综合征(cfs)' + 慢性疲劳症 + 慢性疲劳综合症 | 4544 |
| 2 | 抑郁 + 抑郁症 + 抑郁量表 + 抑郁情绪 + 抑郁症状 + HAMD + BDI + SDS + PHQ-9 | 204052 |
| 3 | 随机 + 随机对照试验 | 235700 |
| 4 | 1 AND 2 AND 3 | 186 |

**The search strategy (Wan Fang)**

| Search Number | Query | Results |
| --- | --- | --- |
| 1 | 慢性疲劳 OR 慢性疲劳综合征 OR 慢性疲劳综合征(cfs) OR 慢性疲劳症 OR 慢性疲劳综合症 | 16636 |
| 2 | 抑郁 OR 抑郁症 OR 抑郁量表 OR 抑郁情绪 OR 抑郁症状 OR HAMD OR BDI OR SDS OR PHQ-9 | 722602 |
| 3 | 随机 OR 随机对照试验 | 2874779 |
| 4 | 1 AND 2 AND 3 | 757 |

**The search strategy (VIP)**

| Search Number | Query | Results |
| --- | --- | --- |
| 1 | 慢性疲劳+慢性疲劳综合征+慢性疲劳症+慢性疲劳综合症 | 4396 |
| 2 | 抑郁+抑郁症+抑郁量表+抑郁情绪+抑郁症状+HAMD+BDI+SDS+PHQ | 439,238 |
| 3 | 随机+随机对照试验 | 339,567 |
| 4 | 1 AND 2 AND 3 | 545 |

**The search strategy (Sinomed)**

| Search Number | Query | Results |
| --- | --- | --- |
| 1 | 慢性疲劳 OR 慢性疲劳综合征 OR 慢性疲劳综合征(cfs) OR 慢性疲劳症 OR 慢性疲劳综合症 | 16636 |
| 2 | 抑郁 OR 抑郁症 OR 抑郁量表 OR 抑郁情绪 OR 抑郁症状 OR HAMD OR BDI OR SDS OR PHQ-9 | 2835 |
| 3 | 随机 OR 随机对照试验 | 2066223 |
| 4 | 1 AND 2 AND 3 | 2775 |
